# Data on analysis of temperature inversion during spontaneous combustion of coal

**DOI:** 10.1016/j.dib.2019.104304

**Published:** 2019-07-24

**Authors:** Jun Guo, Hu Wen, Yin Liu, Yongfei Jin

**Affiliations:** a School of Safety Science and Engineering, Xi'an University of Science and Technology, Xi'an, 710054, China; b Key Laboratory of Western Mine and Hazard Prevention, Ministry of Education of China, Xi'an, 710054, China; c State Mine Emergency Rescue (Xi'an) Research Center, Xi'an, 710054, China

**Keywords:** Coal spontaneous combustion, Temperature-programmed experiments, Various index gases, Environmental pollution control

## Abstract

Data in this article presents the characteristic parameters of spontaneous combustion of coal with different ranks, including lignite, long flame coal, and anthracite. The coal samples were tested by the temperature programmed method. The gas concentration data produced at different temperature points during the heating process are obtained. Through monitoring the spontaneous combustion of coal in a coal mine, the field data in goaf are obtained. Through processing on the data from the experiment and field, three gas indices were obtained, which include CO/CO_2_, Graham value and Alkane ratio. The data is made available for further use and for furthering the understanding of the key findings of the related research, such as the early warning for spontaneous combustion of coal. For more insight please see A method for evaluating the spontaneous combustion of coal by monitoring various gases (Guo et al., 2019).

**Table:** Specifications table

Subject area	Environmental Engineering
More specific subject area	Environmental; Chemical; Coal fire, Safety engineering
Type of data	Table, graph, figure
How data was acquired	The data was acquired by the temperature programmed test method in the laboratory and in the field of a coal mine. The gas data of spontaneous combustion of coal were determined by using gas chromatography (SP-2120, made in China).
Data format	Analyzed.
Experimental factors	One anthracite, one lignite, and two long flame coal were selected. Each coal sample was crushed and screened out to 200 g of coal samples with the particle size of 0–0.9 mm, 0.9–3 mm, 3–5 mm, 5–7 mm and 7–10 mm. The samples were mixed into 1000g samples, numbered 1–4, sealed and preserved.
Experimental features	The samples were supplied with 120 ml/min air flow and ventilated at a constant temperature for 30 minutes. The sample temperature was raised at a rate of 0.3 °C/min and a sample of the resulting gases was collected at each 10 °C interval for analysis by chromatography.
Data source location	Xi'an University of Science and Technology, Xi'an, China.
Data accessibility Related research article	The data are available with this article. Jun Guo, Hu Wen, Xuezhao Zheng, Yin Liu, Xiaojiao Cheng A method for evaluating the spontaneous combustion of coal by monitoring various gases Process Safety and Environmental Protection https://doi.org/10.1016/j.psep.2019.04.014

**Table:** 

**Value of the data** • Experimental data from three typical coal (lignite, long flame coal, and anthracite) tested with temperature-programmed experiment method, which is potentially valuable to further study of coal spontaneous combustion. • Experimental data of spontaneous combustion from three ranks coal can not only be used to further study the spontaneous combustion characteristics of different coal grades but also to study the differences among them. • The data from the laboratory and the field of coal mine can provide a lot of high-value data for other scholars to study how to correlate analysis above two types of data, in order to make the test data better used in field engineering practice. • The data, which used in a new comprehensive evaluating method in this paper, could be potentially valuable to accurately determinate the spontaneous combustion of coal.

## 1 Data

The data shared in this paper include the gas data of coal samples acquired by the temperature programmed test, the field data from a coal mine with the spontaneous combustion of the coal seam, and processed data of the above data. The gas data (O_2_, N_2_, CO, CO_2_, CH_4_, C_2_H_6_, C_2_H_4_) of anthracite, lignite and two long-flame coal obtained through experimental tests, as shown in Table 1, Table 2, Table 3, Table 4. The field observation data is shown in Table 5, come from a goaf of coal seam where spontaneous combustion occurred. This coal seam belongs to a coal mine in Shaanxi province. What's more, the three index parameter data (CO/CO_2_, Graham value, chain alkane ratio) calculated from the index gas, as shown in Table 6 and Table 7.

**Table 1:** Experimental data in lignite heating process.

Temperature/^o^c	O_2_/%	N_2_/%	CO/ppm	CO_2_/ppm	CH_4_/ppm	C_2_H_6_/ppm	C_2_H_4_/ppm
30	20.42	79.36	14.83	988.67	6.08	0.88	–
40	20.30	78.91	34.93	1397.20	9.11	1.14	–
50	19.95	78.73	101.20	3264.52	11.11	1.24	–
60	18.54	79.24	257.10	11178.26	11.81	1.46	–
70	16.78	80.31	454.40	18176.00	4.90	1.66	–
80	14.35	82.20	814.00	26258.07	6.44	1.70	3.22
90	14.11	82.82	1024.00	27675.68	9.52	1.79	4.29
100	13.41	83.31	1042.00	27421.05	9.58	1.82	5.42
110	13.89	82.83	1183.00	30333.33	9.61	1.96	5.62
120	10.86	84.47	1725.00	39204.55	13.57	2.54	7.45
130	4.92	88.83	2875.00	55288.46	22.49	3.02	13.90
140	1.93	89.20	4746.00	69794.12	40.88	4.46	17.92
150	1.27	88.38	7227.00	83068.97	69.88	5.48	20.62
160	1.16	88.29	9431.00	91563.11	84.08	6.43	23.30
170	1.13	87.91	11850.00	96341.46	100.70	7.01	23.61

**Table 2:** Experimental data in anthracite heating process.

Temperature/^o^c	O_2_/%	N_2_/%	CO/ppm	CO_2_/ppm	CH_4_/ppm	C_2_H_6_/ppm	C_2_H_4_/ppm
30	20.64	78.61	6.64	2088.68	4.32	2.57	–
40	20.79	78.40	7.91	1869.50	7.96	3.19	–
50	20.83	78.15	8.89	1760.00	8.34	3.57	–
60	20.53	78.22	21.90	2585.60	11.85	4.76	–
70	20.10	78.38	50.80	4673.41	13.24	5.26	–
80	19.44	79.22	94.90	6856.94	15.57	6.44	–
90	18.82	79.51	159.90	9095.56	19.34	9.44	–
100	18.74	79.47	216.40	7781.37	24.78	10.26	–
110	18.81	80.52	310.00	5145.23	27.81	14.56	7.93
120	18.76	80.51	427.40	3810.97	34.16	18.72	9.78
130	18.37	80.61	628.10	3392.20	43.64	22.03	11.87
140	17.66	80.35	955.50	4562.17	56.59	28.89	14.83
150	15.37	82.29	1783.00	8319.72	75.74	33.90	17.53
160	14.27	83.50	2477.00	10250.79	83.15	36.13	22.33
170	11.64	86.38	3732.00	12462.02	95.26	48.93	30.13

**Table 3:** Experimental data in long flame coal (1#) heating process.

Temperature/^o^c	O_2_/%	N_2_/%	CO/ppm	CO_2_/ppm	CH_4_/ppm	C_2_H_6_/ppm	C_2_H_4_/ppm
30	20.85	78.66	6.50	664.15	10.71	4.03	–
40	20.77	78.17	14.62	731.00	10.86	6.08	–
50	20.65	78.40	38.12	953.00	10.89	13.65	–
60	20.61	78.27	58.51	1244.89	12.21	16.68	–
70	20.07	78.31	156.20	2947.17	13.37	18.00	–
80	19.71	79.27	294.00	3500.00	14.58	20.86	–
90	18.42	80.39	526.00	3867.65	14.69	23.35	–
100	17.40	81.85	817.50	5413.91	15.98	23.87	–
110	16.74	81.24	1174.00	5870.00	16.17	24.81	1.03
120	16.47	82.10	1870.00	9303.48	17.10	24.91	1.35
130	17.60	80.64	2106.00	9360.00	18.22	29.39	3.83
140	16.18	80.72	3433.00	13203.85	34.58	31.23	9.24
150	11.43	84.11	9226.00	25310.69	83.14	33.65	13.38
160	10.39	85.07	11450.00	24361.70	123.15	34.81	16.33
170	9.69	85.51	13950.00	27493.64	155.80	36.22	24.25

**Table 4:** Experimental data in long flame coal (2#) heating process.

Temperature/^o^c	O_2_/%	N_2_/%	CO/ppm	CO_2_/ppm	CH_4_/ppm	C_2_H_6_/ppm	C_2_H_4_/ppm
30	20.78	78.73	3.25	160.29	2.37	1.03	–
40	20.30	79.07	11.86	965.80	2.39	1.35	–
50	20.64	78.45	17.18	580.60	2.47	2.31	–
60	20.09	78.76	54.32	1522.42	2.58	2.69	–
70	20.21	79.21	83.07	1560.59	2.63	3.66	–
80	19.65	79.33	187.20	3657.68	2.72	4.06	–
90	18.30	80.02	370.20	6576.66	3.05	4.10	–
100	17.46	80.29	491.90	7264.81	3.42	4.24	1.55
110	16.41	82.70	809.80	6739.91	4.57	4.63	3.09
120	12.47	85.08	1819.00	12781.96	8.51	6.47	4.91
130	10.57	85.75	2859.00	17485.17	13.38	10.45	9.25
140	7.39	88.19	4647.00	25214.32	25.80	15.59	14.30
150	7.24	88.13	6432.00	31400.12	42.99	21.59	24.08
160	5.14	88.58	9150.00	41719.86	86.00	23.94	32.88
170	4.56	89.15	11350.00	47944.92	121.50	25.64	36.77

**Table 5:** The field observation data.

Time/d	O_2_/%	N_2_/%	CO/ppm	CO_2_/%	CH_4_/%	C_2_H_6_/%
1	14.37	74.33	0.00	0.22	8.02	3.01
2	14.26	71.20	0.00	0.24	9.70	4.02
3	14.37	71.65	0.00	0.16	8.63	4.94
4	11.98	73.78	0.00	0.38	8.20	3.82
5	11.59	76.21	0.01	0.33	6.87	4.36
6	12.68	78.01	0.01	0.31	4.84	3.57
7	13.87	77.41	0.01	0.29	4.35	3.60
8	12.93	76.97	0.01	0.37	4.95	4.03
9	14.30	74.40	0.01	0.24	5.68	5.27
10	14.52	74.65	0.01	0.22	5.44	5.14
11	14.73	77.80	0.01	0.19	2.82	3.56
12	14.25	75.70	0.01	0.22	3.98	4.82
13	14.56	76.02	0.01	0.20	4.07	5.04
14	14.32	77.38	0.01	0.24	4.27	3.64
15	13.44	77.10	0.01	0.25	4.61	4.42
16	15.13	76.09	0.01	0.19	4.14	4.12
17	13.35	77.94	0.01	0.22	4.02	3.85
18	14.01	77.11	0.01	0.24	4.24	4.29
19	12.61	78.17	0.01	0.25	4.24	3.66
20	11.67	78.01	0.01	0.26	4.49	5.27
21	11.50	80.10	0.01	0.32	4.14	3.70
22	12.75	76.73	0.01	0.25	4.57	5.38
23	12.05	76.49	0.01	0.24	5.18	5.78
24	12.92	76.06	0.01	0.23	5.13	5.57
25	13.24	77.38	0.01	0.24	3.88	4.56
26	13.61	77.60	0.01	0.23	3.38	4.80
27	12.51	78.22	0.01	0.29	3.85	4.84
28	10.33	79.61	0.01	0.37	4.08	4.51
29	11.75	80.57	0.01	0.35	3.59	3.49
30	12.56	78.73	0.01	0.27	3.95	4.32
31	12.64	79.38	0.01	0.27	3.86	3.66
32	12.78	78.18	0.01	0.23	3.75	4.90
33	10.46	80.05	0.01	0.35	4.19	2.91
34	11.17	81.60	0.01	0.27	3.58	3.20
35	11.03	81.89	0.01	0.45	3.58	2.53

**Table 6:** Calculations results of gas index data for different coal samples.

Temperature/^o^c	Lignite			Anthracite			Long flame coal (1#)			Long flame coal (2#)		
	CO/CO_2_	Graham	Alkane ratio	CO/CO_2_	Graham	Alkane ratio	CO/CO_2_	Graham	Alkane ratio	CO/CO_2_	Graham	Alkane ratio
30	0.015	0.255	0.097	0.003	0.186	0.424	0.010	0.433	0.249	0.020	0.150	0.429
40	0.025	0.499	0.117	0.004	0.371	0.414	0.020	0.636	0.560	0.012	0.168	0.545
50	0.031	0.966	0.160	0.005	0.521	0.397	0.040	1.089	0.929	0.030	0.481	0.898
60	0.023	1.047	0.146	0.008	0.466	0.445	0.047	1.500	1.144	0.036	0.598	1.020
70	0.025	1.077	0.126	0.011	0.565	0.533	0.053	1.680	1.463	0.053	1.052	1.347
80	0.031	1.223	0.182	0.014	0.609	0.463	0.084	2.279	1.916	0.051	1.387	1.331
90	0.037	1.486	0.194	0.018	0.733	0.423	0.136	2.039	2.180	0.056	1.370	1.201
100	0.038	1.373	0.186	0.028	0.960	0.517	0.151	2.271	1.955	0.068	1.389	0.928
110	0.039	1.664	0.147	0.060	1.413	0.457	0.200	2.756	1.553	0.120	1.766	0.544
120	0.044	1.701	0.144	0.112	1.906	0.441	0.201	4.129	1.457	0.142	2.132	0.484
130	0.052	1.788	0.113	0.185	2.390	0.470	0.225	6.194	1.613	0.164	2.740	0.405
140	0.068	2.489	0.074	0.209	2.859	0.520	0.260	7.122	1.271	0.184	3.414	0.363
150	0.087	3.663	0.064	0.214	3.169	0.564	0.365	9.645	0.405	0.205	4.675	0.251
160	0.103	4.754	0.065	0.242	3.681	0.749	0.470	10.792	0.283	0.219	5.770	0.197
170	0.123	5.965	0.064	0.299	3.986	0.914	0.507	12.330	0.232	0.237	6.902	0.175

**Table 7:** Calculation results of gas index data for field monitoring.

Time/d	CO/CO_2_	10 × Graham	Alkane ratio	Time/d	CO/CO_2_	10 × Graham	Alkane ratio
1	0.014	0.467	0.375	19	0.040	1.216	0.863
2	0.015	0.519	0.415	20	0.042	1.168	1.173
3	0.024	0.573	0.572	21	0.036	1.200	0.895
4	0.012	0.521	0.466	22	0.045	1.394	1.177
5	0.016	0.574	0.634	23	0.048	1.318	1.115
6	0.019	0.709	0.738	24	0.053	1.485	1.085
7	0.023	0.939	0.827	25	0.048	1.507	1.177
8	0.020	0.929	0.814	26	0.051	1.611	1.422
9	0.035	1.225	0.927	27	0.042	1.425	1.256
10	0.041	1.389	0.944	28	0.033	1.153	1.103
11	0.053	1.562	1.265	29	0.034	1.265	0.972
12	0.054	1.734	1.213	30	0.043	1.363	1.094
13	0.056	1.738	1.238	31	0.044	1.399	0.949
14	0.042	1.497	0.854	32	0.047	1.338	1.307
15	0.041	1.348	0.958	33	0.032	1.063	0.694
16	0.051	1.636	0.994	34	0.043	1.201	0.896
17	0.039	1.137	0.958	35	0.026	1.183	0.706
18	0.039	1.330	1.011	–	–	–	–

## 2 Experimental design, materials and methods

### 2.1 Experimental design

#### 2.1.1 Sample preparation

**Fig. 1:**
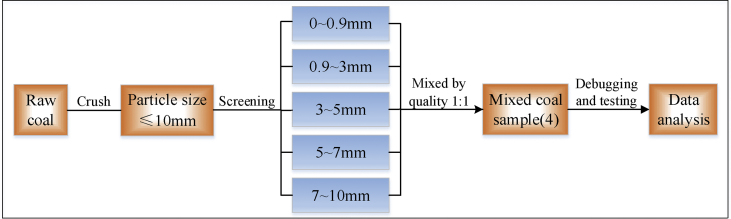
A flow chart summarizing coal sample processing process.

Select lignite, long flame coal, and anthracite, collect fresh exposed coal blocks in working face, crush them in air, sift out 200 g coal samples with particle size of 0–0.9 mm. 0.9–3 mm, 3–5 mm, 5–7 mm and 7–10 mm, mix them into 1000g samples, numbered 1–4#, and prepare for sealed storage test. The sample preparation process is shown in Fig. 1.

#### 2.1.2 Experimental test method

**Fig. 2:**
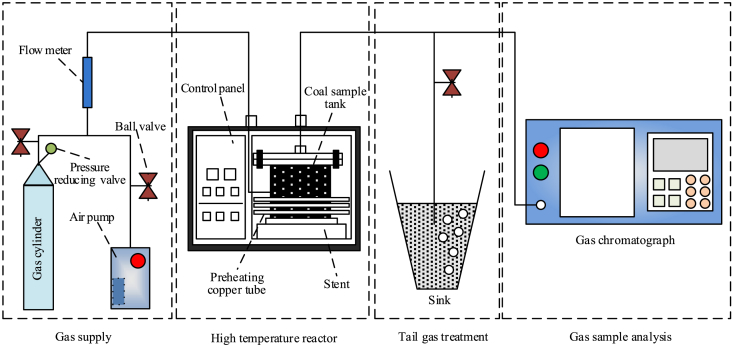
The temperature programmed experimental apparatus.

A temperature programmed test system for spontaneous combustion of coal in air bath is used in the experiment [2], [3], [4]. The structure of the system is shown in Fig. 2. Using this experimental device, coal samples are loaded into a cylindrical special steel coal sample tank with a diameter of 10 cm at the bottom and a height of 25 cm. As shown in Fig. 2, the experiment begins after sealing. Using an air pump or gas cylinder as a gas source, the air is supplied to a coal sample tank with 120 ml/min air flow. Air flows through a glass rotor flowmeter and gas conveying copper pipe, preheated by the heating box, and then passes through the bottom of the coal sample tank to the coal sample. After 30 minutes of venting air, the gas samples were collected at the rate of 0.3 °C/min and then analyzed by the SP-2120 gas chromatograph. The component data of gas products at different temperature points were obtained.

### 2.2 Data analysis

When the experimental data and field data are obtained, the biggest influence factor is the difference in air volume [5], [6], so there are significant differences in the data results. In the analysis of the results, it is necessary to eliminate the dilution effect of air flow on the gas as far as possible. The gas index can satisfy this condition very well [7]. In addition, the mechanism of producing index gas in the coal oxidation process is different, CO and a lot of CO_2_ are produced. It is produced by the oxidation reaction between coal and oxygen. Some coal seams contain a part of CO_2_, CH_4_ and C_2_H_6_ gas, which will be resolved at low temperatures. There is generally no C_2_H_4_ in coal seams. C_2_H_4_ gas is produced mainly by cracking, so different gas means the reaction function represented by the standard is also different [8], [9], [10]. CO/CO_2_ can better reflect the intensity of oxidation, Graham value ($\text{G}=\Delta \text{CO}/\Delta {\text{O}}_{2}$) can reflect the relationship between oxygen consumption and CO formation, and alkane ratio can reflect the intensity of coal pyrolysis [11]. By calculating the experimental and field data, the gas index data are obtained as shown in Table 6, Table 7.

### 2.3 Inversion method of coal temperature

**Fig. 3:**
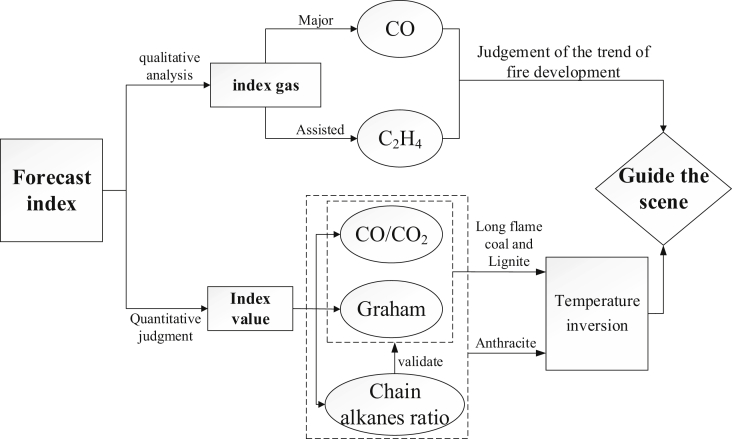
A flow chart summarizing the method of determining coal temperature.

By calculating the results of experimental data and field data, the results can be well corresponded. The natural ignition trend of the coal seam is qualitatively analyzed by selecting standard gas through experiments, and the degree of spontaneous combustion of the coal seam is quantitatively analyzed by gas index. The development law of spontaneous combustion of the coal seam in mine is predicted by combining the two kind of indexes [1]. The forecasting idea is shown in Fig. 3.

As shown in Fig. 3, firstly using a single indicator to qualitatively determine the potential for spontaneous combustion. The CO volume fraction is employed to determine the likelihood of spontaneous combustion. Due to C_2_H_4_ appeared above approximately 110 °C, so its volume fraction is also assessed to qualitatively evaluate the probability of spontaneous combustion. Secondly, using the gas ratio to determine the probability of spontaneous combustion. The CO/CO_2_ ratio, Graham coefficient, and alkyl chain ratio are all determined via on-site measurements. Based on correlations derived from experimental testing, the above ratios are used to find the coal temperature. A comprehensive analysis of single gases such as CO and C_2_H_4_ as well as gas ratios is employed to calculate the coal temperature. The average values of the on-site indicators are calculated so as to obtain the average coal temperature at the site. The prediction accuracy and confidence intervals are set to the desired values. The degree of confidence for each temperature value obtained from the above process is calculated. An application of these data and this early warning for spontaneous combustion of coal can be found in Ref. [1].
